# A Pivotal Role of Hormones in Regulating Cotton Fiber Development

**DOI:** 10.3389/fpls.2019.00087

**Published:** 2019-02-14

**Authors:** Guanghui Xiao, Peng Zhao, Yu Zhang

**Affiliations:** ^1^ Key Laboratory of the Ministry of Education for Medicinal Plant Resources and Natural Pharmaceutical Chemistry, College of Life Sciences, Shaanxi Normal University, Xi’an, China; ^2^ National Engineering Laboratory for Resource Development of Endangered Crude Drugs in the Northwest of China, College of Life Sciences, Shaanxi Normal University, Xi’an, China; ^3^ Zhengzhou Research Base, State Key Laboratory of Cotton Biology, Zhengzhou University, Zhengzhou, China

**Keywords:** gene expression, signaling pathway, cotton, fiber initiation, phytohormone, fiber elongation

## Abstract

Cotton is the main source of renewable fiber in the world and is primarily used for textile production. Cotton fibers are single cells differentiated from the ovule epidermis and are an excellent model system for studying cell elongation, polyploidization, and cell wall biosynthesis. Plant hormones, which are present in relatively low concentrations, play important roles in various developmental processes, and recently, multiple reports have revealed the pivotal roles of hormones in regulating cotton fiber development. For example, exogenous application of hormones has been shown to promote the initiation and growth of fiber cells. However, a comprehensive understanding about phytohormone regulating fiber development is still unknown. Here, we focus on recent advances in elucidating the roles of multiple phytohormones in the control of fiber development, namely auxin, gibberellin, brassinosteroid, ethylene, cytokinin, abscisic acid, and strigolactones. We not only review the identification of genes involved in hormone biosynthetic and signaling pathways but also discuss the mechanisms of these phytohormones in regulating the initiation and elongation of fiber cells in cotton. Auxin, gibberellin, brassinosteroid, ethylene, jasmonic acid, and strigolactones play positive roles in fiber development, whereas cytokinin and abscisic acid inhibit fiber growth. Our aim is to provide a comprehensive review of the role of phytohormones in cotton fiber development that will serve as the basis for further elucidation of the mechanisms by which plant hormones regulate fiber growth.

## Introduction

Upland cotton (*Gossypium hirsutum*) is one of the most important economic crops, and the fiber produced by this crop is the raw material for the textile industry worldwide. Naturally grown cotton plants are perennial woody shrubs and trees, yet cultivated cotton is mostly an annual crop. Each cotton fiber is a single, phenomenally elongated cotton seed coat epidermal cell. The differentiation and development of cotton fiber cells is a very complicated process and is divided into four overlapping stages: fiber initiation (3 days before anthesis to 2 days post anthesis (DPA), 2–3 DPA), fiber cell elongation (0–25 DPA), cell wall thickening (20–45 DPA) and fiber cell maturation (45–50 days post anthesis) (Basra and Malik, 1984). In recent years, genomics, functional genomics, and proteomics studies have been done to show that ethylene and pectin as well as genes involved in synthesis of secondary cell wall promote fiber growth in cotton (Ji et al., 2003; Shi et al., 2006; Gou et al., 2007; Pang et al., 2010).

Phytohormones, namely gibberellin acid (GA), auxin, cytokinin, brassinosteroid (BR), abscisic acid (ABA), ethylene, jasmonic acid (JA), salicylic acid, and strigolactone (SL), are small endogenous signaling molecules in plants (Davière and Achard, 2016). Many of these hormones have been shown to be directly involved in plant growth, cell elongation, and cell expansion. The analysis of endogenous hormone levels in bolls and fibers and the effects of treating ovules with exogenous hormones indicate that phytohormones are responsible for the development of fiber cells in cotton (Kim and Triplett, 2001; Ahmed et al., 2018). Although the receptors and signaling pathways of these hormones have been identified in *Arabidopsis* in the past two decades, it is still unknown how the components of phytohormone signaling pathways participate in fiber cell development. Taking advantage of the whole genome sequence of cotton (Paterson et al., 2012; Wang et al., 2012; Li et al., 2014, 2015a,b; Zhang et al., 2015), the study of cotton fiber growth has progressed rapidly and has led to new discoveries about the hormones involved in the development of fiber cells. Our manuscript comprehensively and specifically reviews the vital roles of various phytohormones in regulating cotton fiber development, including gibberellin acid, auxin, cytokinin, brassinosteroid, abscisic acid, ethylene, jasmonic acid, and strigolactone. Compared with the review paper (Ahmed et al., 2018), our manuscript also discusses the important role of jasmonic acid and strigolactone during fiber cell development.

## Auxin is Responsible for Both Fiber Cell Initiation and Elongation

Auxin plays a crucial role in various developmental processes, such as plant root development, apical dominance, embryogenesis, vascular differentiation, and the response to internal and external stimuli (Dubrovsky et al., 2008; Mashiguchi et al., 2011; Rakusova et al., 2015; Wang et al., 2015a,b; Xi et al., 2016; Liu et al., 2012). There is also evidence that auxin plays an important role in cotton fiber development. For example, supplementation of exogenous indole-3-acetic acid (IAA) can compensate for defects in fiber elongation (Jasdanwala et al., 1977). Furthermore, exogenous application of IAA, one of the most important natural auxins, significantly increases the total fiber volume (Beasley and Ting, 1974; Guinn and Brummett, 1988; Chen and Guan, 2011). Consistent with its role in fiber development, auxin begins to accumulate before flowering, peaking around 2–3 DPA, and gradually decreases to the initial level at 10 DPA (Chen and Guan, 2011). Furthermore, driving expression of the IAA biosynthetic gene *iaaM* under the control of the petunia MADS box gene *FBP7* (*Floral Binding protein 7*) promoter in the epidermis from −2 DPA to 10 DPA cotton ovules was found to significantly enhance fiber cell initiation at the ovule epidermis and greatly increase the number of lint fibers, which finally resulted in a greater than 15% increase in fiber yield (Zhang et al., 2011).

Recent studies have shown that auxin in fiber cells is mainly derived from outside of the ovule rather than from *in situ* synthesis. Auxin is transported from the ovules into the fibroblasts by the PIN-FORMED polar auxin transporters (GhPINs). The flow of auxin in fiber cells and the establishment of hormone gradients in epidermal cells of the ovule are primarily mediated by the GhPIN3a protein. *GhPIN3a* gene is highly expressed in the outer cells of 0 DPA ovules. Ovule-specific inhibition of multiple *GhPIN* genes significantly inhibits fiber cell initiation and elongation (Zhang et al., 2016). The *GhPIN1a_Dt*, *GhPIN6_At*, and *GhPIN8_At* genes were preferentially expressed in the fiber initiation and elongation stages; these genes also increase the density and length of leaf trichomes, which are organs similar to fiber cells (Zhang et al., 2017a,b). These results indicate that GhPIN-mediated auxin transport plays an important role in fiber-specific auxin accumulation in cotton, which should be further verified by genetic evidences.

Besides the biosynthesis and transport pathways, the auxin signaling pathway also contributes to cotton fiber cell development. Five putative auxin response genes are highly expressed during fiber cell elongation (6–12 DPA) (Gou et al., 2007). GhARF2 and GhARF18, two auxin response factors, are highly expressed during fiber cell initiation, and overexpression of these two genes significantly promotes trichome initiation in *Arabidopsis* leaves (Xiao et al., 2018). These data imply that GhARF2 and GhARF18 are positive regulators of cotton fiber cell initiation. In contrast to *GhARF2* and *GhARF18*, *INDOLEACETIC ACID-INDUCED PROTEIN 16* (*GhIAA16*) may play a negative role in fiber initiation and elongation. *GhIAA16* is relatively lowly expressed in wild-type ovules. However, in the *fuzzless-lintless* (*fl*) mutant, *GhIAA16* transcripts are the most highly expressed immediately after flowering (Han et al., 2012).

Expression profiling has provided further evidence that auxin plays an essential role in fiber cell elongation. The expression level of the auxin binding protein *GhABP* was found to increase by about 59-fold from 0 to 10 DPA. Further analyses have shown that *GhABP* is only expressed in elongated fibroblasts but not in villous mutants and undifferentiated epidermal cells (Chen et al., 2001). These results suggest that *GhABP* may be involved in the elongation of cotton fiber cells. Although *ABP* is essential for growth and development in *Arabidopsis*, the biological function of *GhABP* in cotton is still unknown. Overexpression and knockout of *GhABP* gene in cotton should be performed to study the function of *GhABP* gene in the future. Rac, a small G protein, mediates intracellular auxin signal transduction (Wu et al., 2011). To date, several *Rac* genes have been identified in cotton. *GhRac1* is highly expressed during fiber elongation, and expression gradually decreases when secondary wall biosynthesis in fiber cells begins (Kim and Triplett, 2004). *GhRacA* and *GhRacB* were found to be widely expressed in roots, stems, leaves, hypocotyls, and fiber cells, with the highest transcription level observed in fiber cells at the initiation and elongation stages (Li et al., 2005). GhMAPK, a member of MAPK family involved in auxin signal pathway, is predominately expressed in elongated fiber cells (Chen et al., 2001). All members of AGAMOUS subfamily were significantly induced by auxin (Moura et al., 2016). The expression levels of most GhSAUR genes are responsive to exogenous application of IAA treatment (Li et al., 2017).

Auxin also plays an important role in fiber cell wall thickening stage. Based on the activities of IAA oxidase and superoxide enzymes, the level of IAA at the secondary wall thickening stage was four times than that in fiber cells during elongation (Singh et al., 2009), suggesting that auxin may regulate the expansion of the primary fiber cell wall and the synthesis of the secondary wall. The exogenous application of 1-naphthaleneacetic acid (NAA), an analogue of auxin, further confirmed that auxin is involved in the synthesis of secondary wall cellulose (Zhang et al., 2011).

**Figure 1:**
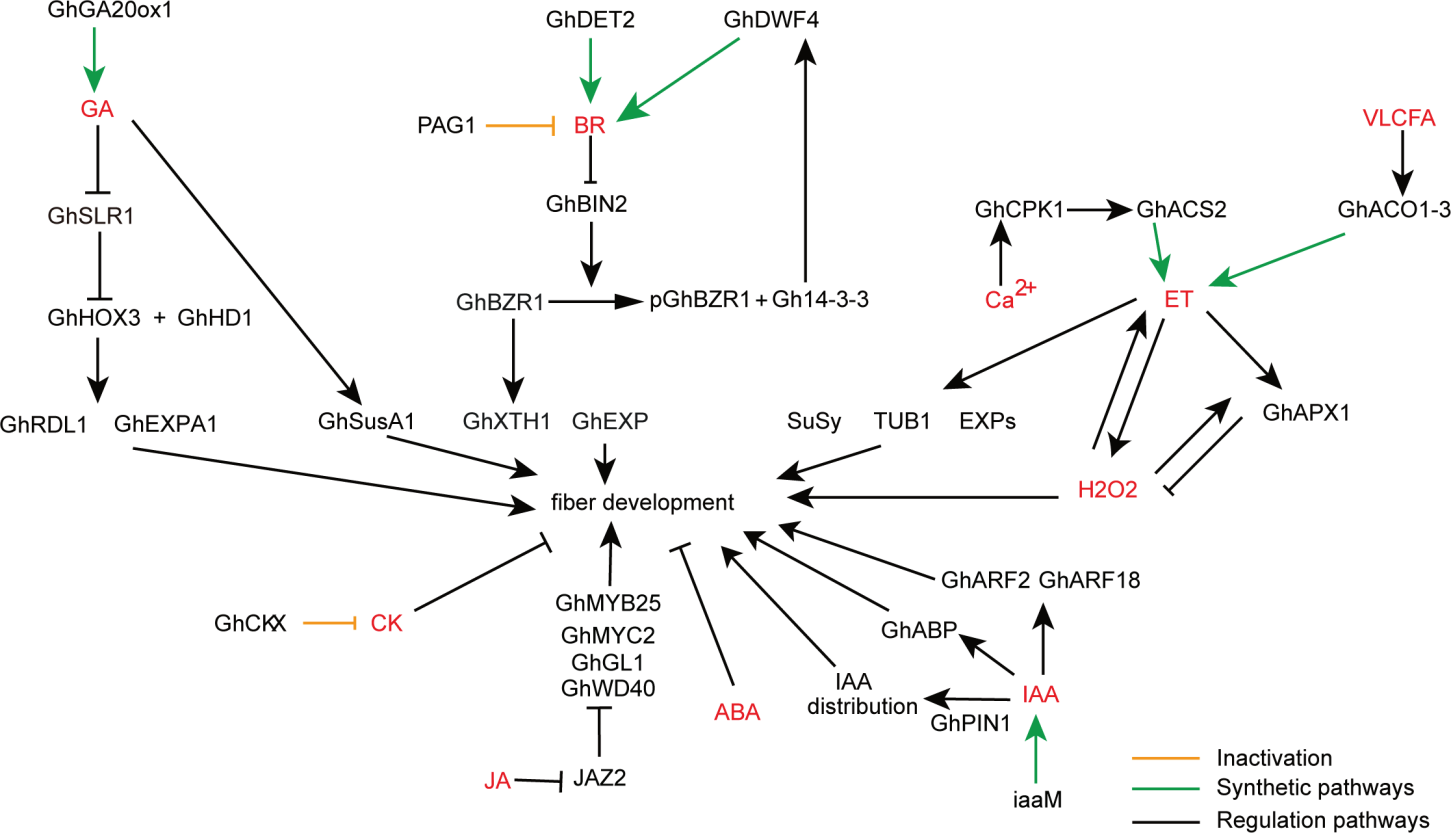
A schematic model showing the roles of various phytohormones during cotton fiber development. Arrows show promotion actions and bars ends show inhibitory actions. Yellow lines indicate inactivation pathway, green lines indicate synthetic pathway, and black lines indicate regulation pathway. GA, gibberellic acid; BR, brassinosteroids; VLCFA, very long-chain fatty acid; ET, ethylene; H_2_O_2_, hydrogen peroxide; CK, cytokinin; ABA, abscisic acid; JA, jasmonic acid; IAA, indoleacetic acid.

Taken together, these findings suggest that auxin plays critical roles in fiber cell initiation and elongation in cotton (Figure 1). More genetic experiments need to be done to further confirm this conclusion by overexpressing and knocking out key genes involved in auxin synthesis and signaling pathways.

## Gibberellic Acid Contributes to The Elongation of Cotton Fibers

GA is a biologically active diterpene hormone that regulates seed germination, stem elongation, epidermal hair development, and fruit development (Huang et al., 2015; David et al., 2016). Previous studies have shown that exogenous application of GA significantly promotes cotton fiber elongation (Kim and Triplett, 2001). However, when the GA synthesis inhibitor paclobutrazol was added into the ovule culture medium, there were fewer and shorter fibers than in the absence of treatment (Liao et al., 2009). The application of gibberellic acid increased the IAA and ABA content during fiber development stage and also improved fiber strength, micronaire, and maturation in natural-colored cottons (Zhang et al., 2017a,b). GA accumulation is also correlated with cotton fiber elongation. The content of GA increases rapidly after flowering, reaching a peak in 10 DPA fiber cells, and then decreases quickly (Xiao et al., 2010). The endogenous level of GA_3_, a bioactive form of GA, in long-staple cotton varieties is significantly higher than that in medium- and short-fiber varieties (Aleman et al., 2008). In addition, overexpression of the gibberellin 20-oxidase1 (GhGA20ox1) in transgenic cotton, which significantly increased the content of two bioactive forms of GA, GA_3_ and GA_4_, resulted in the production of more and longer fibers (Xiao et al., 2010).

DELLA proteins, which are negative regulators of the GA signaling pathway, interact with transcription factors or key regulatory proteins to inhibit their binding to target genes or their transcriptional activation activities (Davière and Achard, 2013). When GA is present, it binds to the receptor GID1 to initiate the ubiquitin-mediated degradation of DELLA proteins, releasing key transcription factors that trigger the expression of GA-responsive genes (Sun, 2008; Davière and Achard, 2016). The cotton DELLA proteins GhGID1 and GhSLR1 are specifically expressed in fiber cells, and GhGID1 interacts with GhSLR1 in response to GA. Ectopic expression of GhSLR1 in *Arabidopsis* results in a dwarf plant phenotype and enhances the transcription of GA-responsive genes (Aleman et al., 2008; Dong et al., 2009).

In order to reveal the mechanisms by which GA promotes cell elongation, more genes responsive to GA have been identified. *Xyloglucan endotransglycosylase/hydrolase* (*XTH*) and *expansin* (*EXP*) genes involved in promoting cell elongation are induced by GA (Aleman et al., 2008). The transcript levels of the sucrose synthase gene *GhSusA1* and sucrose synthase activity are significantly higher in transgenic fiber cells overexpressing GA 20-oxidase than in wild-type fiber cells (Bai et al., 2014). In addition, exogenous application of bioactive GA enhances the transcription of *GhSusA1* in both fiber cells and hypocotyls (Bai et al., 2014). These results suggest that GA promotes secondary cell wall development in cotton fiber cells by regulating the expression of sucrose synthase genes (Figure 1). Recently, a new mechanism for the promotion of cotton fiber elongation by GA was identified. The transcription factor GhHOX3 is a core regulator in the GA signaling pathway (Shan et al., 2014). When the level of GA is low, the DELLA protein GhSLR1 specifically interacts with GhHOX3 and prevents GhHOX3 from regulating its target genes. However, when the level of GA is high, the GhSLR1 protein is degraded and releases GhHOX3. GhHOX3 then interacts with GhHD1 and promotes the expression of the two cell wall genes, *GhRDL1* and *GhEXPA1*.

## Brassinosteroids Play an Important Role in Fiber Initiation and Elongation

BRs are a class of plant steroid hormones involved in a variety of physiological and developmental processes, including cell division and elongation, vascular tissue differentiation, reproductive development, senescence, and tolerance to biotic and abiotic stress (Anuradha and Rao, 2001; Haubrick and Assmann, 2006; Ming et al., 2008). BRs are also important for cotton fiber cell development. In cotton, application of a low concentration of brassinolide, a bioactive BR isolated from plants, significantly promotes fiber cell elongation, whereas brassinazole (BRZ), a BR biosynthesis inhibitor, significantly inhibits fiber cell development *in vitro* (Sun et al., 2005). Exogenous application of BRZ to cotton floral buds leads to severe defects in fiber cell differentiation. Steroid reduction, catalyzed by steroid 5α-reductase (DET), is considered a major rate-limiting step in BR biosynthesis. GhDET2, a cotton steroid 5α-reductase, is highly expressed during fiber cell initiation and elongation, and silencing of *GhDET2* transcription inhibits both fiber cell initiation and elongation (Ming et al., 2008). Application of finasteride, a steroid 5α-reductase inhibitor, also significantly inhibits fiber elongation, which can be restored by applying BR. Overexpression of *GhDET2* driven by the seed coat-specific promoter *pFBP7*, increases both fiber number and length. *GhSMT1*, another gene important for sterol biosynthesis, is highly expressed in 10 DPA fiber cells (Shi et al., 2006). *GhPAG1*, a homolog of *Arabidopsis CYP734A1* involved in BR biosynthesis, controls cotton fiber development by regulating the level of endogenous BRs (Yang et al., 2014). The cotton *pag1* mutant has reduced fiber length, and fiber elongation can be restored by BR treatment. Taken together, these findings suggest that BR is required for both fiber cell initiation and elongation (Figure 1). Importantly, the biological functions of *GhBZR1* and *GhBES1* genes need to be further clarified during cotton fiber development.

The components in BR signaling pathway are found to be involved in fiber cell development. BRASSINOSTEROID INSENSITIVE 1 (BRI1), which encodes a plasma membrane-localized leucine-rich repeat receptor kinase, is a BR receptor (Peng et al., 2018). The cotton, *GhBRI1* gene is predominantly expressed in the fiber cells at the elongation stage and completely complements the phenotype of the *Arabidopsis bri1–5* mutant (Sun et al., 2004, 2005). In *Arabidopsis*, BRASSINOSTEROID-INSENSITIVE 2 (BIN2), a key negative regulator in BR signaling pathways, regulates root epidermal cell fate specification by phosphorylating ENHANCER OF GLABRA 3 (EGL3) and TRANSPARENT TESTA GLABRA 1 (Guo et al., 2013; Dubrovsky et al., 2008; Mashiguchi et al., 2011; Rakusova et al., 2015; Wang et al., 2015a,b; Xi et al., 2016; Liu et al., 2012). *Arabidopsis* plants overexpressing *GhBIN2* rescue the reduced growth resulted from the loss of function of *BIN2* gene (Sun and Allen, 2005). BRASSINAZOLE-RESISTANT 1 (BZR1), a core transcription factor in the BR signaling pathway, regulates the transcription of its target genes by binding to a BRRE box in the promoter region (Guo et al., 2013). 14-3-3 proteins interact with BZR1 and affect its nucleocytoplasmic shuttling in response to BR signaling (Gampala et al., 2007). In cotton, overexpression of *Gh14-3-3 L* promotes fiber elongation, resulting in an increase in the length of mature fibers, and silencing of *Gh14-3-3 L* significantly reduces the initiation and elongation of fiber cells (Zhou et al., 2015). This short-fiber phenotype can be partially restored by exogenous application of BR. Further analyses have shown that *Gh14-3-3 L* can interact with GhBZR1 and that the 14-3-3-regulated GhBZR1 protein can directly bind the *GhXTH1* and *GhEXP* promoters to regulate gene expression during the fiber cell elongation stage (Zhou et al., 2015). Recently, a newly identified gene, GhbHLH282, is found to not only regulate fiber development but also involve in BR signaling (Lu et al., 2018). Interestingly, there is no report about GhBZR1 regulation of cotton fiber growth due to the severe sterility resulting from the overexpression of *GhBZR1* gene in cotton.

## Ethylene Regulates Fiber Cell Elongation

The phytohormone ethylene plays a pivotal role in plant growth, regulating processes such as root hair development, hypocotyl growth, and apical hook formation (Dubois and Van den Broeck Inzé, 2018). Ethylene biosynthesis consists of two steps: S-adenosylmethionine is converted into 1-aminocyclopropane-1-carboxylic acid (ACC), which is catalyzed by ACC synthases (ACS). ACC is further converted into ethylene, which is catalyzed by ACC oxidase (ACO) (Yang et al., 2015). Ethylene promotes root hair growth by regulating ethylene insensitive 3 (EIN3) and root hair defective (RHD6) activity in *Arabidopsis* (Feng et al., 2017). Recently, cellulose synthase-like D3 (*AtCSLD3*) in *Arabidopsis* and *GhCSLD3* in cotton were found to act downstream of ethylene to mediate root growth and cell elongation (Hu et al., 2018).

In cotton, application of ethylene *in vitro* significantly promotes the elongation of fiber cells, and the ethylene synthesis inhibitor L-(2-aminoethoxyvinyl)-glycine (AVG) inhibits fiber cell elongation (Shi et al., 2006). The ethylene biosynthesis pathway is one of the most significant biochemical pathways during fiber elongation stage. *GhACO1-3* is significantly expressed during this stage, which is consistent with the high ethylene production in elongating fiber cells. The transcripts of three *GhACO* genes were found to specifically accumulate in 10 DPA fibers. Recently, excessive levels of *ACO1* and *ACO3* transcripts and ethylene were found to accumulate in *Gossypium raimondii*, resulting in the abortion of fiber cells (Li et al., 2015a,b). Ca^2+^-dependent protein kinase 1 interacts with and phosphorylates cotton ACS2, which increases its activity and results in a significant increase in ethylene production (Wang et al., 2011). Conversely, the production of an appropriate amount of ethylene in *Gossypium hirsutum* was found to significantly promote fiber development. Interestingly, ethylene can restore the inhibition of fiber growth resulting from the application of BRZ, whereas BR does not restore the shortening of fibers caused by application of AVG. In addition, ethylene promotes the expression of genes encoding inositol synthase, tubulin, and expansion, which are all involved in cotton fiber development (Shi et al., 2006).

Ethylene may also promote fiber elongation by promoting the production of hydrogen peroxide (H_2_O_2_), a type of reactive oxygen species (ROS) that significantly promotes fiber cell elongation *in vitro* (Qin et al., 2008). Ascorbate peroxidase (APX) is an ROS-scavenging enzyme that is involved in the regulation of intracellular ROS levels (Jiang et al., 2017). Compared with ovules in the *fl* mutant, *GhAPX1* is highly expressed in five DPA fiber cells in wild-type cotton (Li et al., 2007). Exogenous application of H_2_O_2_ significantly induces the transcription of *GhAPX1* and enhances APX activity (Qin et al., 2008). Exogenous ethylene promotes the production of H_2_O_2_ during fiber elongation, indicating that the H_2_O_2_-induced cotton fiber elongation may occur downstream of the ethylene signaling pathway. Overexpression of the calmodulin *GhCaM7* also promotes fiber elongation, whereas *GhCaM7* RNAi plants have delayed fiber initiation and inhibited fiber elongation (Tang et al., 2014). In contrast to wild type, *GhCaM7*-overexpressing fiber cells have increased ROS content and *GhCaM7* RNAi fibers have significantly reduced ROS accumulation, suggesting that GhCaM7 promotes cotton fiber cell elongation by regulating ROS production. Interestingly, H_2_O_2_ can increase the influx of Ca^2+^ into fiber cells.

Saturated very long-chain fatty acids (VLCFAs) also promote the production of ethylene and thus regulate cotton fiber growth (Qin et al., 2007). Ethylene effectively eliminates the inhibition of fiber cell elongation resulted from the application of 2-chloro-N-[ethoxymethyl]-N-[2-ethyl-6-methyl-phenyl]-acetamide (ACE), the inhibitor of VLCFA biosynthesis, whereas VLCFA cannot overcome this inhibition caused by AVG. Application of C24:0 fatty acids *in vitro* results in a significant increase in *ACO* transcripts, resulting in significant ethylene production. Therefore, ROS regulates the accumulation of Ca2+, which then promotes fiber elongation, likely by promoting ethylene production. Besides, VLCFA can promote ethylene biosynthesis to enhance fiber cell elongation (Qin and Zhu, 2011).

## Cytokinin Contributes to the Development of Cotton Ovules, but not Fiber Cells

Cytokinin regulates many aspects of plant development, such as cell division, senescence of plant tissues and organs, and apical dominance (Liu et al., 2017). There is evidence that cytokinin plays a role in ovule development. The addition of exogenous cytokinins into ovule culture medium significantly promotes the growth of ovules but inhibits the elongation of fiber cells (Beasley et al., 1974). This may be due to the fact that rapid cell division occurs during embryo development, but only single cells expand during fiber cell elongation. The cytokinin compound kinetin (6-furfurylaminopurine) significantly increases the yield of cotton seed *in vitro* (Sawan et al., 2000). However, large-scale commercial applications of cytokinins are not practically feasible in crops because of high costs and the time required (Li et al., 2004). The accumulation of endogenous cytokinin is mainly in wild-type ovules, but not in fiber cells (Chen et al., 1997). Cytokinin is present at a relatively low concentration in unfertilized ovules, but levels continue to increase after anthesis (Beasley et al., 1974).

Genetic modification is a good strategy for manipulating the concentration of hormones such as cytokinin and investigating the resulting phenotypes. Cytokinin levels can be increased by overexpressing isopentenyl transferase (IPT), a rate-limiting enzyme involved in cytokinin biosynthesis (Spallek et al., 2017). Overexpression of the *GhIPT* gene in cotton under the control of the CaMV35S promoter or a seed-specific promoter significantly increases the accumulation of cytokinin but has no impact on fiber yield and quality (Zhu et al., 2018). Cytokinin oxidase/dehydrogenase (CKX), which catalyzes the cleavage of the unsaturated side chain of cytokinin N6 and leads to the loss of cytokinin activity, is an important negative regulator of cytokinin metabolism (Niemann et al., 2018). Inhibition of CKX expression can increase endogenous levels of cytokinin in plants. Silencing of *GhCKX* transcripts in transgenic cotton plants using RNAi technology resulted in a significant increase in seed number and slightly enhanced the yield of fibers (Jones and Schreiber, 1997; Zhao et al., 2015). In conclusion, we suggest that cytokinin is mainly required for seed development and plays a negative role in fiber cell growth in cotton (Figure 1). Both seed development and fiber growth need to be investigated in the transgenic cottons overexpressing the *GhCKX* gene.

## Abscisic Acid Inhibits the Initiation of Cotton Fibers

ABA is mainly involved in seed dormancy and stress response (Tuan et al., 2018). Previous studies have revealed that *in vitro* application of ABA not only inhibits fiber cell elongation but also inhibits the initiation of cotton fibers (Beasley et al., 1974). This inhibition is highly correlated with an increase in ABA levels. When measuring the content of endogenous ABA in different fiber cells, it was found that the accumulation of ABA gradually increased during the fiber cell initiation and elongation stages (0–10 DPA), decreased during the period of rapid elongation (10–20 DPA), and then returned to the original low level during the maturation stage (30–50 DPA) (Davis and Addicott, 1972). The ABA in fibers around 16 DPA will undergo a transition, which is consistent with the formation of the secondary wall of fiber cells (Yang et al., 2001). This indicates that ABA may be involved in secondary wall biosynthesis. The ABA content in short-staple fibers is much higher than that in long-staple fibers throughout development (Nayyar et al., 1989). Moreover, levels of endogenous ABA in cotton ovules are positively correlated with the short fiber production (Ma et al., 2011). Much higher accumulation of ABA in 0 DPA ovules was observed in the short fiber cotton mutant *Ligon-lintless 1* than in wild type (Gilbert et al., 2013). The promoter of GbEXPA2, a cotton fiber-preferential promoter, can be regulated by both GA and ABA (Li et al., 2015a,b). Taken together, these results indicate that ABA seems to be a negative regulator of cotton fiber initiation (Figure 1), which should be further confirmed by investigating the number of fibers per ovule in the transgenic cottons overexpressing ABA synthetic genes or in cotton with high ABA content.

## Jasmonic Acid Positively Controls Fiber Cell Initiation in Cotton

JA, which is an important endogenous hormone, is biosynthesized from linolenic acid *via* the octadecanoid pathway (Jia et al., 2015). Linolenic acid is oxygenated by lipoxygenase (LOX) to generate a peroxide, which is then converted into oxophytodienoic acid, a precursor of JA, by allene oxide synthase (AOS). Although its roles in the response to abiotic and biotic stress are well known, JA also plays a pivotal role in regulating aspects of plant growth and development, including growth inhibition, trichome development, leaf abscission, and senescence (Delker et al., 2006). For example, JA significantly enhances the number of trichomes in leaves (Traw and Bergelson, 2003). In contrast to wild-type plants, *aos* mutant plants have leaves with significantly fewer trichomes, and this defect can be rescued by exogenous application of JA (Yoshida et al., 2009). JA significantly promotes the expression of *GL3*, a key bHLH transcription factor involved in trichome formation, and increases trichome initiation. The Jasmonate-ZIM domain (JAZ) proteins are negative regulators of JA signaling (Song et al., 2011). JAZ interacts with multiple members of the WD-repeat/bHLH/MYB transcriptional complex, including GL1, GL3, and EGL3, which are key components involved in trichome growth, to repress trichome initiation (Qi et al., 2011). In the absence of JA, JAZ inhibits the expression of GL1, GL3, and EGL3, and in the presence of JA, JAZ is degraded by the 26S proteasome, thus releasing these transcription factors and promoting trichome initiation.

In cotton, JA-associated metabolism contributes to cotton fiber initiation (Wang et al., 2015a,b). GhJAZ2 is highly expressed in ovules at the fiber initiation stage, and overexpression of GhJAZ2 inhibits fiber initiation and reduces the length of fiber cells (Hu et al., 2016). GhJAZ2 can interact with the GhMYB25-like, GhGL1, GhMYC2, and GhWD40 proteins, which are the core components of the WD-repeat/bHLH/MYB transcriptional complex and are reported to be involved in fiber development (Figure 1).

Taken together, a number of genes related to phytohormone biosynthesis and signaling pathway have been found to be involved in cotton fiber cell development (Table 1). Other functions of these genes should be the focus of future research in cotton.

**Table 1:** Genes involved in phytohormone synthesis as well as signaling pathway and cotton fiber development.

Gene	Method	Up/downregulated	Transgenic/non-transgenic
GhGA20ox1	PCR	Upregulated	Transgenic
GhSLR1	PCR	Upregulated	Transgenic
GhHOX3	PCR	Upregulated	Transgenic
GhHD1	PCR	Upregulated	Transgenic
GhRDL1	PCR	Upregulated	Non-transgenic
GhEXPA1	PCR	Upregulated	Non-transgenic
GhDET2	PCR	Upregulated	Transgenic
GhDWF4	PCR	Upregulated	Transgenic
GhARF2	PCR	Upregulated	Non-transgenic
GhARF18	PCR	Upregulated	Non-transgenic
Gh14–3-3	PCR	Upregulated	Transgenic
GhAPX1	PCR	Upregulated	Non-transgenic
GhBIN2	PCR	Upregulated	Non-transgenic
GhXTH1	PCR	Upregulated	Non-transgenic
GhEXP	PCR	Upregulated	Non-transgenic
GhPIN1	PCR	Upregulated	Non-transgenic
GhMYB25	PCR	Upregulated	Non-transgenic
GhMYC2	PCR	Upregulated	Non-transgenic
GhGL1	PCR	Upregulated	Non-transgenic
GhWD40	PCR	Upregulated	Non-transgenic
GhSusA1	PCR	Upregulated	Transgenic
GhBZR1	PCR	Upregulated	Non-transgenic
GhACO1–3	PCR	Upregulated	Non-transgenic
GhSuSy	PCR	Upregulated	Transgenic
GhTUB1	PCR	Upregulated	Non-transgenic
GhABP	PCR	Upregulated	Non-transgenic
GhJAZ2	PCR	Downregulated	Transgenic
GhPAG1	PCR	Downregulated	Transgenic
GhCPK1	PCR	Upregulated	Transgenic
GhACS2	PCR	Upregulated	Transgenic
GhCKX	PCR	Downregulated	Transgenic

## Perspectives

Although auxin, GA, BR, ethylene, and JA have been shown to promote fiber cell development in cotton and a number of genes have been reported to be involved in cotton fiber cell development (Ahmed et al., 2018), a large number of questions still remain to be answered. Auxin polar transporters (PINs) and the core regulators of the auxin signaling pathway (ARFs) play important roles in regulating cotton fiber development (Zhang et al., 2017a,b), but the mechanisms of auxin transport between the ovule and fibers and the targets of ARF proteins are still a mystery. In the future, transgenic technology can be used to generate transgenic cotton with PIN overexpression and knockout and to study the distribution of auxin in ovules and fibers of transgenic cotton. Exploring and identifying target genes of GhARF2 and GhARF18 is also a very effective way to study the molecular mechanism of auxin signaling pathway regulating fiber development in cotton. Exogenous application of BR promotes cotton fiber development, and overexpression of the BR biosynthetic gene *GhDET2* significantly increases the length of fiber cells (Sun et al., 2005; Ming et al., 2008). However, how the BR signaling pathway regulates cotton fiber development and the roles of BZR1 and BRI1-EMS-SUPPRESSOR 1 (BES1), two core factors in BR signaling pathway, are still unknown. We have too little understanding of how JA regulates fiber growth. Identification of downstream target genes of GhMYB25-like, GhGL1, GhMYC2, and GhWD40 to elucidate JA signaling pathway will help us better understand the molecular mechanism of JA regulation of fiber development. The interactions between key regulators of different hormone signaling pathways will reveal the cross talk between various phytohormones involved in fiber initiation and elongation.

Recently, another class of plant hormones, SL, has attracted scientists’ attention, and the SL signaling pathway is becoming more and more clear (Khosla and Nelson, 2016). SL was first isolated from cotton roots in 1966 (Cook et al., 1966), and more recent studies have revealed that SL mainly functions as an inhibitor of shoot branching (Jiang et al., 2013; Zhou et al., 2013; Yao et al., 2016). Interestingly, SL was found to regulate root hair elongation in *Arabidopsis* (Kapulnik et al., 2011a,b). The cross talk between SL, ethylene, and auxin may be essential for the elongation of root hairs (Kapulnik et al., 2011a,b), which are similar to cotton fiber cells. Although the biological function of SLs and the SL signaling pathway have been extensively studied in *Arabidopsis*, the roles of SLs during cotton development, especially during fiber cell growth, are still unknown. Whether SL is involved in cotton fiber development is key question and will be a topic of research in the future. The relationship between SL and fiber development can be investigated by overexpressing or knocking out SL biosynthetic and signaling pathway genes and then examining the lengths of cotton fiber cells.

The key genes of biosynthesis and signaling pathways of these hormones have been known in *Arabidopsis*. Our review summarizes some important members of these pathways, which contribute to cotton fiber development, and also prompts us to investigate whether the remaining key components of these pathways regulate fiber cell growth to further characterize the mechanisms of plant hormones regulating fiber growth.

## Author Contributions

GX and ZP write the manuscript, ZY revised the manuscript. All authors read and approved the final manuscript.

### Conflict of Interest Statement

The authors declare that the research was conducted in the absence of any commercial or financial relationships that could be construed as a potential conflict of interest.
